# Evaluation of buprenorphine as optimisation of postoperative analgesia in feral cats undergoing ovariohysterectomy under field conditions

**DOI:** 10.1111/jsap.70064

**Published:** 2025-12-03

**Authors:** V. Heitzmann, A. Diggelmann, E. Goldinger, A. Schiele

**Affiliations:** ^1^ Section of Anaesthesiology, Department of Clinical Services and Diagnostics, Vetsuisse Faculty University of Zurich Zürich Switzerland; ^2^ Tiermedizinisches Zentrum AG Müllheim Germany

## Abstract

**Objectives:**

The objective of this prospective, randomised, blinded, observational clinical study was to investigate the effect of subcutaneously administered buprenorphine on postoperative pain in female feral cats undergoing ovariohysterectomy as part of a trap‐neuter‐return programme. The aim was to determine whether buprenorphine can prevent pain scores from exceeding established intervention thresholds postoperatively – a problem identified in a previous study using the same anaesthetic protocol.

**Materials and Methods:**

A total of 115 cats were anaesthetised with an intramuscular protocol comprising medetomidine, ketamine and butorphanol, in combination with a nonsteroidal anti‐inflammatory drug (meloxicam). Prior to anaesthesia induction, cats were randomly assigned to either receive 0.02 mg/kg buprenorphine (BUP group) or an equal volume of saline (NaCl 0.9%) (control group) subcutaneously at the end of surgery. Pain was assessed at 3, 6 and 24 hours postoperatively using the validated feline Glasgow Composite Measure Pain Scale.

**Results:**

Subcutaneous administration of buprenorphine at the end of surgery did not significantly reduce postoperative pain scores or prevent values from exceeding the clinical intervention threshold compared to saline. Although numerical differences were observed at specific time points, pain trajectories over time were similar between groups. Notably, overall and independently of the group 34.5% of cats exceeded the intervention threshold at 3 hours and 38.8% at 6 hours postoperatively, underscoring the need for improved analgesic strategies during the early postoperative period in trap–neuter–return settings.

**Clinical Significance:**

These findings suggest that a single subcutaneous dose of buprenorphine may not consistently enhance analgesia over standard multimodal protocols under field conditions.

## INTRODUCTION

Spay‐neuter programmes are increasingly implemented as a mandatory strategy to control the overpopulation of stray cats (Sparkes et al., 2013). Effective pain management is essential to uphold animal welfare, particularly in high‐volume trap‐neuter‐return (TNR) initiatives where thousands of free‐roaming cats are sterilised within a short timeframe (Looney et al., 2008). In such programmes, animals are typically returned to their original environment within 24 hours after surgery, often without the opportunity for extended monitoring, structured pain assessment or repeated administration of analgesics. This presents a significant challenge to provide adequate postoperative care – especially regarding pain management, which is a cornerstone of responsible surgical practice (Mathews et al., 2014).

Multimodal analgesia, the use of drugs with different mechanisms of action, is widely recommended to achieve effective and balanced postoperative pain control. The World Small Animal Veterinary Association (WSAVA) endorses this approach even for routine procedures such as ovariohysterectomy in cats (Monteiro et al., 2023). However, under field conditions, logistical and financial constraints often limit the implementation of advanced or prolonged analgesic protocols.

Opioids remain a fundamental component of systemic analgesia. Buprenorphine, a partial μ‐opioid receptor agonist, is commonly used in feline medicine due to its favourable safety profile (rarely severe respiratory depression, minor cardiovascular effects, long duration without accumulation with repeated administration, wide therapeutic index) and relatively long duration of action (4 to 8 hours) (Steagall et al., 2014). Its ease of administration and relatively low cost make it particularly well suited for use in field conditions or private practices, where continuous monitoring and redosing may not be feasible (Robertson, 2008; Steagall & Monteiro, 2019). Despite these advantages, nonsteroidal anti‐inflammatory drugs (NSAIDs) are often the only analgesics administered postoperatively, typically for several days. However, recent evidence suggests that this approach may not align with the actual time course of postoperative pain in cats (Heitzmann et al., 2022; Steagall & Monteiro, 2019).

In a previous field study using a standard injectable anaesthetic protocol (medetomidine, ketamine, butorphanol) combined with a single NSAID dose, more than one‐third of cats exceeded the clinical pain threshold 6 hours after surgery (Heitzmann et al., 2022). Notably, pain scores returned to baseline by 20 hours postoperatively, indicating that the peak of acute surgical pain occurs within the first few hours following ovariohysterectomy. This raises an important welfare concern: a significant proportion of animals may experience unalleviated acute postoperative pain during the critical hours immediately after surgery, at a time when they are often already being prepared for release. Addressing this acute pain phase is therefore of utmost importance and could be achieved by adding analgesics, such as buprenorphine, to the existing anaesthesia and NSAID protocol. Its duration of efficacy (4 to 8 hours) matches the critical postoperative window and provides a practical, effective solution under field conditions (Steagall et al., 2014).

The objective of this study was to determine whether subcutaneous (sc) administration of buprenorphine, as an adjunct systemic analgesic, would reduce postoperative pain in female feral cats undergoing ovariohysterectomy in the context of a large‐scale TNR programme. We hypothesised that cats receiving buprenorphine would exhibit significantly lower pain scores at 6 hours after surgery and that fewer individuals would exceed the clinical cut‐off score for intervention compared to those receiving the standard anaesthetic and NSAID protocol alone.

## MATERIALS AND METHODS

### Study design

This prospective, randomised, blinded, observational study was conducted within a trap‐neuter‐return (TNR) programme for feral cats in North Cyprus, supported by the Network for Animal Protection (NetAP). Cats were captured and handled in accordance with NetAP's animal welfare standards and all procedures were performed by experienced veterinary professionals following these guidelines (www.netap.ch). The study protocol received ethical approval from the relevant institutional animal care and use committee from North Cyprus. Anaesthesia and postoperative pain assessments were performed and monitored by personnel trained in veterinary anaesthesia, including residents of the European College of Veterinary Anaesthesia and Analgesia (ECVAA).

### Animals

A total of 115 feral cats (61 females, 54 males) of unknown age were surgically sterilised, with females undergoing ovariohysterectomy and males castration. All cats included in the study were adults based on body size, dentition and overall physical appearance. Cats were captured from local colonies using commercial live traps and transported to the surgical facility the evening prior to surgery. Each cat was housed individually in numbered cages of identical size and setup (including blankets), within a quiet, temperature‐controlled room to minimise environmental variability during postoperative pain assessment.

Upon arrival, all cats were observed from a distance to evaluate general health and identify any overt signs of illness or pathology. Because a full physical examination was not always feasible due to the feral nature of the animals, only cats that appeared clinically healthy on visual inspection were enrolled in the study and only female cats were included in the treatment arm evaluating postoperative pain following ovariohysterectomy. Early pregnancy was defined as the presence of small, non‐distended uterine horns or early embryonic vesicles without visible abdominal enlargement, whereas late pregnancy referred to advanced gestation with obvious uterine distension or palpable fetal structures. Late‐pregnant cats were excluded, either preoperatively or, if detected only intraoperatively, at that stage. Shortly before the induction, the cats were transferred to a closed room, placed in a restraint cage on a scale and then weighed by subtracting the weight of the cage. Each cat’s body condition score was defined using the AAHA scale (Cline et al., 2021).

### Anaesthesia and surgery

Food was withheld for 12 to 14 hours prior to anaesthesia, whereas free access to water was provided. All cats initially received an intramuscular (im) injection of 20 μg/kg medetomidine (Medetor, 1 mg/mL; Cevizli Mahallesi, Tugay Yolu Caddesi, No: 67 Kat: 1, Maltepe, Istanbul, Türkiye), 5 mg/kg ketamine (KETAMIN 10%, 100 mg/mL; Dutch Farm International BV Nieuw Walden 112, Nederhorst den Berg, Netherlands) and 0.4 mg/kg butorphanol (Nalgosed 10 mg/mL; Bioveta a.s, Komenského, Czech Republic). Depending on the depth of sedation/anaesthesia achieved, a supplemental dose of medetomidine and/or ketamine was administered as needed to place a venous catheter. The catheter was aseptically placed in the cephalic vein to establish intravenous (iv) access. To ensure adequate anaesthesia, propofol (Propofol‐PF 1%, 10 mg/mL; Polifarma, Vakiflar OSB Mah. Sanayi Cad. No 22/1, Turkey) was titrated to effect iv in cats that showed a reaction to surgical stimulus (increase in respiratory rate, increase in heart rate or movement) during surgery. Emergency drugs and equipment, including lidocaine spray (2%), endotracheal tubes of various sizes and a laryngoscope, were readily available in case intubation or resuscitation became necessary.

Cats exhibiting physiological abnormalities during preoperative assessment (e.g. severe upper or lower respiratory disease) or intra‐abdominal pathologies (e.g. haemoabdomen) were excluded from the study.

Following anaesthetic induction, all cats received sc administration of 2.5 mg/kg metoclopramide (Metoklopramid hidroklorür 5 mg/mL; Turktipsan, Bügdüz Mahallesi Kaymakam Ali Galip Caddesi, Turkey) as a prophylactic measure to reduce the risk of nausea, vomiting and regurgitation, 0.2 mg/kg meloxicam (Melosyn 5 mg/mL; IPM, Tepeören, Itosb Mh. 3. Cad. No: 7, Turkey) and 15 mg/kg amoxicillin (Syvamox LA 150 mg/mL; Laboratorios Syva S.A., Av. Parroco Pablo Diez 57, Léon, Spain). Preoperative assessment and anaesthesia were performed and monitored by trained anaesthetists (resident and board‐eligible anaesthetist ECVAA).

All cats received supplemental oxygen via facemask at a flow rate of 3 L/minute using an oxygen concentrator. Monitoring parameters included continuous peripheral oxygen saturation (SpO_2_) and heart rate (HR) using a multiparameter monitor (Carescape ONE, Anandic Medical Systems AG, Switzerland), as well as respiratory rate (RR) and rectal body temperature measured with a digital thermometer. Female cats underwent ovariohysterectomy via a ventral midline approach (linea alba) in dorsal recumbency, performed by a single highly experienced surgeon. Throughout surgery, isotonic crystalloid fluids were administered iv at 15 mL/kg/hour. This relatively high rate was intentionally selected to compensate for the short duration of the surgical procedure.

### Study groups

The cats were randomly allocated to the buprenorphine (BUP) or the control (NaCl) group by drawing lots with treatment. Cats in group BUP received 0.02 mg/kg of buprenorphine (Bupaq, 0.3 mg/mL; Streuli, Uznach, Switzerland) and cats in group NaCl received the same volume of 0.9% saline sc at the end of the surgery.

### Postoperative monitoring

In the recovery area, all animals received sc injections of 5.7 mg/kg praziquantel (Anipracit, 56.8 mg/mL; Bavet, ITOSB, 12. Cad. No: 8, Tepeören, Tuzla, Istanbul, Turkey) and 1 mg/kg doramectin (Dectonexx, 10 mg/mL; Alke Saglik Ürünleri, Merkez Organize Sanayi Bölgesi 2. Kisim, 10 Cad. No: 7, Turkey) for endoparasite control. For ectoparasite treatment, 7.5 mg/kg fipronil was applied topically as a spot‐on (Fiprojin 10%, 100 mg/mL; TeknoVet, Zafer, Istanbul, Turkey). If the cats had not lifted their heads within 1 hour of the end of the surgery, 0.1 mg/kg atipamezole (Antisedan, 5 mg/mL; Zoetis Fatih Sultan Mehmet, Istanbul, Turkey) was administered im.

Pain assessments were conducted at 3, 6 and 24 hours postoperatively (measured from buprenorphine injection administered at final skin suture) using the feline Glasgow Composite Measure Pain Scale (CMPS). All assessments were performed by the same observer – an ECVAA anaesthesia resident – who had experience in feline pain assessment and was blinded to the treatment allocation. Each cat was first observed for 1 minute in its cage. To complete the CMPS, the cat was gently encouraged to move, allowing the observer to assess posture, movement and behaviour. The incision site and surrounding abdominal area were then palpated using a long wooden stick to evaluate pain response. Sedation levels were scored using the sedation scale described by Grint et al. (Wagner et al., 2017) adapted to cats.

At the 6‐hour timepoint, food intake was assessed by offering each cat four pieces of commercial food. Consumption was evaluated both immediately and several minutes later, once the cat was undisturbed, to minimise the influence of human presence.

Cats were also monitored for any abnormal clinical signs throughout the postoperative period. If no abnormalities were observed, animals were returned to their original geographical location after the final pain assessment at 24 hours postoperatively.

### Statistical analysis

Statistical analyses were performed in R (version 4.4.3) using the lme4 package for generalised linear mixed models, the geepack package for generalised estimating equations and the base stats package for Poisson regression. All data were assessed for distribution and scale prior to modelling using the Shapiro–Wilk test in addition to visual inspection with boxplots. Visual inspection (boxplots) and statistical tests confirmed that the pain scores were ordinal and not normally distributed, overdispersed data. Therefore, a series of non‐parametric and generalised linear models were applied.

Pain score distributions at each time point were visualised using boxplots, and within‐individual trajectories were plotted to assess the course of postoperative pain across time (3 to 6 to 24 hours). The change in pain over time was calculated for each animal (Δ 6 to 3 hours and 24 to 6 hours) and medians were compared between groups using the Mann–Whitney U test. To assess clinical relevance, the number of cats exceeding a defined pain threshold (total score ≥5) was calculated for each group at every time point.

A generalised linear mixed model (GLMM) with a negative binomial distribution and log link was fitted to model the total pain score as the dependent variable. The model included treatment group (BUP *versus* NaCl), time point (3, 6, 24 hours) and their interaction as fixed effects. Animal ID was included as a random effect to account for repeated measures. Additional covariates were included: weight, body condition score (BCS), combined ketamine/medetomidine dose, propofol dose, surgery time, anaesthesia time, sedation score, pregnancy and atipamezole administration.

To evaluate whether any specific question in the pain scale (1 to 7) was affected by buprenorphine, individual Poisson regression models were fitted for each item score, using treatment group as the independent variable. Cluster‐robust standard errors were used to account for repeated measures within cats.

## RESULTS

During the project a total of 115 feral cats (61 female, 54 male) were anaesthetised with the medetomidine–ketamine–butorphanol mixture. A total of 53 female cats were initially enrolled in the study. Four cats were subsequently excluded: two due to missing pain score data, one due to misidentification as female and one following the incidental intraoperative discovery of a haemoabdomen requiring emergency splenectomy. An additional four cats were found to be in early pregnancy; however, as this did not result in a more invasive surgical procedure and time was comparable to the other cases, they were not excluded from the analysis. Late‐pregnant animals were already tried to be selected out during the selection phase and if this had not been noticed, the cat would have been excluded. Finally, 49 female cats (25 cats (51%) in the buprenorphine group and 24 (49%) in the control group) completed the study.

Body weights of the cats enrolled in the study ranged from 1.4 to 5.5 kg (2.8 ± 0.8 kg; mean ± standard deviation) and the body condition score ranged between 3 and 6/9 (4 ± 0.9; median ± standard deviation). As the age of the cats was not known, this was not included in the statistical analysis. The total injection dose of ketamine and medetomidine ranged from 0.02 to 0.038 mg/kg for medetomidine (0.021 ± 0.004 mg/kg; mean ± standard deviation) and 5 to 9.6 mg/kg for ketamine (5.44 ± 1.05 mg/kg; mean ± standard deviation) across the study population. No cat had to be intubated due to apnoea. The duration from anaesthesia induction to recovery ranged between 18 and 61 minutes (34 ± 10.1 minutes; mean ± standard deviation). The duration of surgery ranged between 6 and 24 minutes (9.4 ± 3.6 minutes; mean ± standard deviation). Nine cats received 0.1 mg/kg of im atipamezole because they failed to lift their heads within 1 hour after anaesthesia induction. There were no significant differences between the groups in terms of body weight (*P* = .74), medetomidine and ketamine dosages (*P* = .39), atipamezole administration (*P* = .73), surgery duration (*P* = .26) or anaesthesia time (*P* = .31).

**FIG 1:**
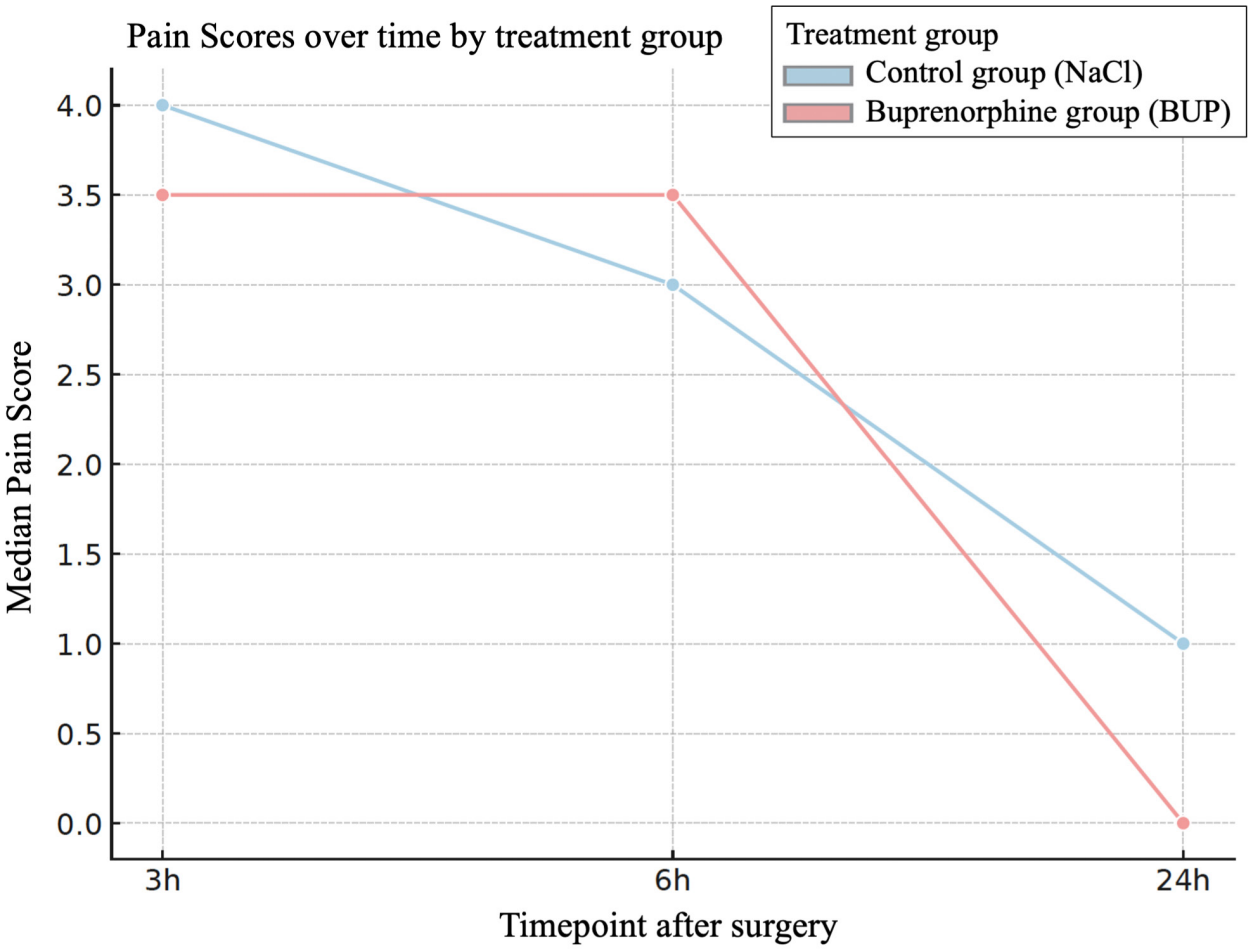
Median pain scores over time (3, 6 and 24 hours), shown separately for the control group and the buprenorphine group.

Pain scores peaked at 3 hours postoperatively (median = 4.0), remained elevated at 6 hours (median = 3.0) and decreased significantly by 24 hours postoperatively (median = 0.5; *P* < .001). The median change in pain score from 3 to 6 hours was zero in the control group and −1 in the buprenorphine group (*P* = .24). Between 6 and 24 hours postoperatively, scores dropped in both groups (NaCl: median −3; BUP: median −2), with no significant group difference (*P* = .23, Fig 1).

The proportion of cats exceeding the clinical pain threshold (≥ 5) was 34.5% at 3 hours (NaCl: 9/25; BUP: 8/24), highest with 38.8% at 6 hours postoperatively (NaCl: 9/25; BUP: 10/24) and declined sharply by 24 hours (NaCl: 2/25; BUP: 3/24, 8.7%), without statistical significance between the groups (Table 1).

**Table 1:** Number of cats that would qualify for rescue analgesia (mild to moderate pain) based on modified Glasgow Composite Pain Scale (mGCPS) performed 3, 6 and 24 hours after the surgical procedure

Timepoint	Total percentage	Buprenorphine	NaCl
3 hours	34.5%	8/24	9/25
6 hours	38.8%	10/24	9/25
24 hours	8.7%	3/24	2/25

**FIG 2:**
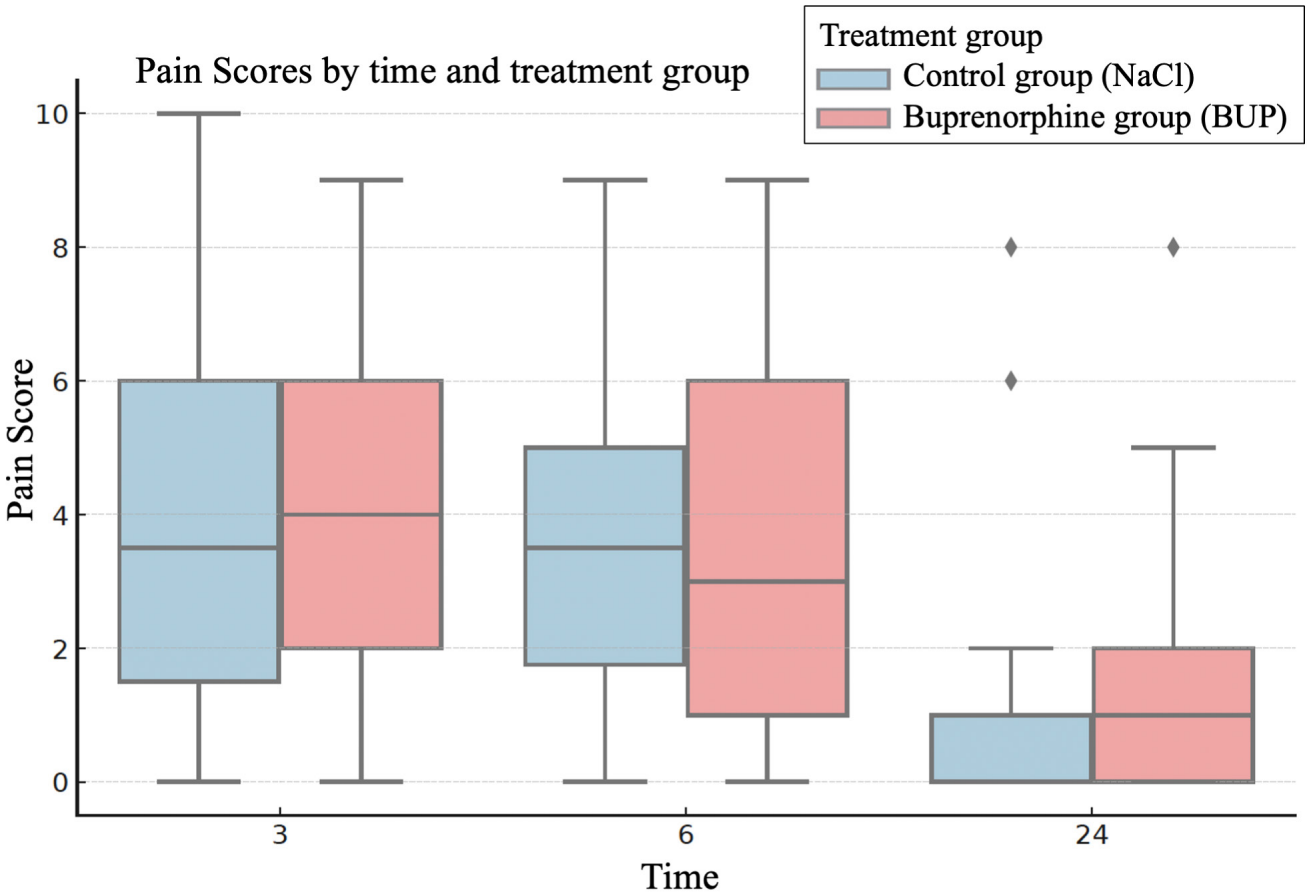
Boxplots of total pain scores in feral female cats at 3, 6 and 24 hours postoperatively, shown separately for the control group and the buprenorphine group. The median scores are indicated by horizontal lines within each box and group distributions overlap across all time points.

In the full GLMM including group, time, interactions and covariates, only the 24 hours time point showed a statistically significant reduction in pain compared to 3 hours postoperatively (*P* = .001). No significant effect of treatment group (*P* = .82), nor group times time interaction (6 hours: *P* = .35; 24 hours: *P* = .64), was found (Fig 2). This indicates that pain scores did not differ between groups at any measured time point, and the change in pain over time followed a similar pattern in both groups. The only covariate approaching significance was anaesthesia duration (*P* = .06), with longer anaesthesia times being associated with higher pain scores. A reduced model including only group, time and their interaction confirmed these findings: pain significantly decreased at 24 hours, but group assignment had no measurable impact at any time point.

A more detailed Poisson regression in which the individual questions (1 to 7) of the pain scale were tested for significance between the groups also showed no difference (Q1 *P* = 1.0, Q2 *P* = .42, Q3, *P* = 1.0, Q4a *P* = .58, Q4b *P* = .88, Q5 *P* = .69, Q6 *P* = .31, Q7 *P* = .21).

A generalised estimating equation (GEE) model with Poisson distribution and log‐link function was applied to evaluate the effects of treatment group, time and pain score on sedation score. The analysis revealed that only the pain score had a statistically significant effect on sedation score (*P* = .04), indicating a positive association: higher pain scores were significantly associated with higher sedation scores. In contrast, the treatment group (*P* = .86) and time did not show a significant influence on sedation score within this model.

## DISCUSSION

The results of this study showed no statistically relevant benefit of adding sc buprenorphine to a standard field anaesthetic protocol (medetomidine, ketamine, butorphanol and meloxicam) for postoperative pain control in feral female cats undergoing ovariohysterectomy. Pain peaked at 3 hours postoperatively in both groups, with approximately one‐third of cats in each group exceeding the established pain score threshold (≥ 5), indicating insufficient analgesia during the acute postoperative phase. This confirms previous findings using similar protocols. Pain scores dropped significantly by 24 hours in both groups, returning to near‐baseline values and confirming previous results in cats and dogs (Heitzmann et al., 2022; Iwe et al., 2024).

The findings highlight that the commonly used field anaesthetic protocol, even when supplemented with an NSAID, does not consistently provide adequate analgesia during the early postoperative period. In privately owned cats, perioperative administration of NSAIDs is common and sometimes continued after surgery (Sandersen, 2023). However, results from this and previous studies suggest that prolonged postoperative treatment may not be necessary, as pain levels typically decrease markedly within 24 hours. Instead, the critical period requiring effective analgesia is the early postoperative phase. Consequently, future protocols should prioritise interventions that provide optimal efficacy during this early phase rather than extending treatment beyond clinically relevant pain duration.

Buprenorphine, although widely recommended for feline analgesia, did not significantly improve pain control when given subcutaneously. This may reflect limitations of its pharmacokinetics in cats, where absorption is often delayed and variable, resulting in inconsistent plasma concentrations (Steagall et al., 2014). Reported sc bioavailability ranges from 30% to 55%, which may have reduced systemic drug availability in some individuals, resulting in insufficient plasma concentrations. Peak plasma levels may not be reached until 1.5 to 2 hours post‐injection, and analgesic effects are less predictable than with im or iv routes. By contrast, im administration typically results in peak effects within 30 to 60 minutes and provides analgesia lasting 6 to 8 hours (Bortolami & Love, 2015; Steagall et al., 2013), a profile that may be more suitable for managing acute postoperative pain.

Additionally, residual effects of concurrently administered butorphanol for induction may have influenced the analgesic response due to its role as a partial μ‐opioid receptor agonist and κ‐agonist. Pharmacokinetic studies in cats have shown that im administration of butorphanol results in a plasma half‐life of approximately 2.5 to 3.5 hours, with antinociceptive plasma concentrations (>45 ng/mL) lasting around 2 to 3 hours, suggesting that receptor‐level effects could still influence pain perception beyond the sedative window (Wells et al., 2008).

However, threshold studies in cats showed that the threshold was only reduced for up to 90 to 120 minutes (Robertson, Lascelles, et al., 2003; Robertson, Taylor, et al., 2003). If μ‐receptor blockade had been the cause, an effect of buprenorphine would have been expected after 6 hours; however, no significant difference was observed at that time point, making it unlikely that butorphanol had any meaningful influence.

Another potential approach to improve postoperative analgesia in feral cats could be the use of long‐acting or encapsulated buprenorphine formulations. These preparations maintain stable plasma concentrations for up to several days but are currently licensed only in the United States (Doodnaught et al., 2017). Their application in large‐scale TNR programmes is limited by cost, market availability and storage requirements, yet they may become a valuable welfare tool as accessibility improves.

Given the inherent variability in individual pain expression – especially in non‐domesticated cats where pain assessment is more challenging – the individual pain trajectories may be more informative than group score comparisons. Although by analysing individual patterns a trend toward improvement in the buprenorphine group at 6 hours was noted, the effect did not reach statistical significance. Given the pharmacokinetics of buprenorphine, peak effects would be expected within 1 to 2 hours; therefore, a reduction in pain should have been evident at 3 hours. However, delayed and variable absorption after sc administration, together with possible residual butorphanol receptor occupancy, may have postponed or attenuated its analgesic effect (Steagall et al., 2013).

Future studies should evaluate administering buprenorphine at induction via the im and/or higher doses to ensure adequate plasma concentrations and better align its peak effect with the period of greatest postoperative pain, thereby avoiding the use of butorphanol in the anaesthetic protocol.

The variability of individual pain scores also underscores that a uniform regimen does not suit all individuals, especially in high‐volume TNR programmes where monitoring is limited (Levy et al., 2003; Wallace & Levy, 2006). By mimicking realistic field conditions, this study characterised the natural course of postoperative pain and identified the critical window in which analgesic coverage is insufficient. The absence of a rescue analgesia protocol was aimed at maximising the likelihood of detecting potential analgesic gaps when using a protocol commonly applied for feral cat neutering. Nevertheless, although no specific cut‐off pain score was predefined, any cat exhibiting ethically unacceptable levels of pain, as judged by the assessors, while the cats were still under veterinary care, would have received appropriate analgesic intervention. This approach enabled an accurate evaluation of the number of cats and the duration of their experience of postoperative pain. These findings therefore provide a necessary foundation for the future optimisation of analgesic strategies, allowing more precise pharmacological targeting of the identified postoperative window. The high proportion of animals exceeding clinical cut‐off values highlights the importance of postoperative pain scoring to detect insufficient analgesia. However, pain scoring in feral cats presents unique challenges, as it can be difficult to distinguish between behavioural signs of pain, fear and stress (Cambridge et al., 2000; Reid, Scott & Nolan, 2017).

Furthermore, the survival instincts of feral cats, including suppression of pain‐related behaviours to avoid appearing vulnerable, likely lead to underestimation of discomfort. This behavioural restraint complicates pain evaluation, as subtle signs can be missed in stressful environments such as field clinics, where fear‐related behaviours like immobility or crouching may overlap with pain indicators.

Despite these limitations, structured and systematic pain assessment remains indispensable as it helps identify animals needing additional analgesia and provides insights to refine future pain management strategies in field settings. The consistent detection of elevated pain scores during the acute postoperative phase across multiple individuals and studies strongly supports the sensitivity and validity of the Glasgow Composite Measure Pain (CMPS, Reid, Scott, Calvo et al., 2017) in recognising clinically relevant pain, even in feral cats under field conditions where behavioural expression is often subdued. These repeated findings highlight the importance of applying validated pain scoring systems to maintain and improve feline welfare standards. In the study, pain scoring was refined through minor methodological adjustments. For safety reasons, palpation around the wound was performed using a wooden stick and enabled reliable assessment of the surgical site even when the cat was in sternal recumbency. The stick was carefully introduced ventrally along the abdomen, and gentle dorsal pressure was applied to the incision area, providing a consistent and central (ventral) access to the wound region. To minimise observer bias, only the modified Glasgow Composite Measure Pain Scale (mGCPS) was used, avoiding a second scale that could influence scoring. All assessments were performed by a single trained observer, though multiple blinded observers could have improved reliability under these challenging conditions. A significant correlation between longer surgical duration and higher postoperative pain scores supports the scale’s construct validity in detecting increased nociception associated with more invasive procedures. However, sedation may have influenced scoring, as behaviours like crouched posture or reluctance to move can reflect either sedation or pain, risking misclassification – especially in feral cats, where behavioural nuances are harder to interpret (Steagall & Monteiro, 2019).

Limitations of this study include several methodological and contextual constraints.

While the mGCPS is validated for use in domestic cats, it has not been specifically validated for feral or non‐socialised individuals as discussed above. Adaptations were necessary for safety reasons – for example, the use of a wooden stick for palpation to maintain distance – potentially altering the standardised application of the scale.

Another limitation was the lack of thorough preoperative clinical examination and the absence of baseline pain scoring, both of which were not feasible under field conditions. Stress factors associated with confinement, transport and unfamiliar surroundings would likely have elevated behavioural indicators of fear and arousal, thereby confounding any attempt to establish a true baseline reflecting pain‐free behaviour. Moreover, prolonged acclimatisation before surgery was not ethically or logistically possible within the context of a high‐throughput TNR programme, as it would have increased the duration of captivity and associated stress. Nevertheless, obtaining a baseline might have provided valuable information to better interpret the individual trajectory of postoperative pain scores and to mitigate potential confounding factors. Consequently, omitting baseline assessment was considered the most welfare‐friendly and scientifically justified approach under these field conditions.

The timing of the first pain assessment (3 hours post‐surgery) was based on pharmacokinetic data suggesting that the effects of ketamine (half‐life ~1 hour; Baggot & Blake, 1976), medetomidine (sedation duration ~60 to 90 minutes; Salonen, 1989) and butorphanol (antinociception 90 to 120 minutes; Robertson, Lascelles, et al., 2003; Robertson, Taylor, et al., 2003) would have mostly subsided by that point. Butorphanol’s antagonist to partial agonist activity at μ‐opioid receptors may sustain beyond the sedative window; therefore, lingering receptor interactions and alterations of buprenorphine’s efficacy cannot be entirely excluded. However, the likelihood of a clinically relevant impact appears low, as no significant analgesic benefit of buprenorphine was observed from 6 hours postoperatively onward, when sedative effects would be expected.

Subcutaneous buprenorphine (0.02 mg/kg), when added to a medetomidine–ketamine–butorphanol anaesthetic protocol with meloxicam, did not significantly improve postoperative analgesia in feral cats undergoing ovariohysterectomy. Pain scores peaked at 3 hours and declined by 24 hours in both groups and over one‐third of cats exceeded the rescue threshold in the early postoperative period. These findings highlight the urgent need to optimise analgesic protocols in routine neutering, particularly in TNR settings where ongoing monitoring is not feasible. A targeted study is needed to evaluate whether, without butorphanol, a higher sc dose or the im administration of buprenorphine can provide effective analgesia for up to 6 hours and to characterise pain score trajectories over 8, 10, 12 hours and beyond – data currently lacking.

## Author contributions

V.H. designed the study. V.H., A.D., E.G. and A.S. performed the surgeries and collected field data. V.H. carried out the statistical analysis. V.H. prepared the first draft of the manuscript. A.D., E.G. and A.S. critically revised and approved the final version of the manuscript.

## Conflict of interest

The authors declared no potential conflicts of interest with respect to the research, authorship and/or publication of this article.

## Funding

Drugs were sponsored by NetAP Switzerland.

## Supporting information


Data S1:

## Data Availability

The data that supports the findings of this study is available in the supplementary material of this article.
